# Feed intake of the sow and playful creep feeding of piglets influence piglet behaviour and performance before and after weaning

**DOI:** 10.1038/s41598-019-52530-w

**Published:** 2019-11-06

**Authors:** Anouschka Middelkoop, Natasja Costermans, Bas Kemp, J. Elizabeth Bolhuis

**Affiliations:** 1 Adaptation Physiology Group, Department of Animal Sciences, Wageningen University & Research, P.O. Box 338, 6700 AH Wageningen, The Netherlands; 2 Human and Animal Physiology Group, Department of Animal Sciences, Wageningen University & Research, P.O. Box 338, 6700 AH Wageningen, The Netherlands

**Keywords:** Animal physiology, Animal behaviour

## Abstract

Creep feed intake is variable and may be partly homeostatically and exploratory driven. We studied effects of maternal feed restriction and a ‘play-feeder’ on piglet behaviour and performance. 37 Litters received creep feed in a conventional (CON) or play-feeder (PL) and their sows were full-fed (FF) or restrictedly-fed (RES). Eaters were determined via rectal swabs. At weaning (d24) four piglets from the same treatment were grouped (n = 36 pens). RES hindered piglet growth by 41 g/d and enhanced time eating, creep feed intake and percentage of eaters at weaning versus FF. RES-PL had the largest proportion of moderate and good eaters. PL stimulated feeder exploration and attracted more piglets to the feeder than CON. Post-weaning, RES increased exploratory behaviours, feed intake between d0–5, and growth between d0–2, and reduced body lesions between d0–2 (within CON), drinking and ear biting. PL increased ingestive behaviours, feed intake and growth between d0–15, and BW at d15 post-weaning by 5%. PL also lowered the prevalence of watery diarrhoea, number of body lesions and piglets with ear (within FF) and tail (within RES) damage at d15 post-weaning. Treatments did not affect FCR. To conclude, RES and particularly PL (broader and for longer) result in less weaning-associated-problems.

## Introduction

The transition from feeding on sow’s milk to foraging and feeding exclusively on solid feed, i.e. weaning, is a gradual process occurring between 8.5 and 22 weeks of age in free range pigs^1,2^. In commercial farms, weaning occurs earlier and sudden by separation of the sows and their piglets, generally at 3 to 4 weeks of age in Europe. Apart from this nutritional challenge, as piglets are abruptly not able to suckle anymore, weaning also simultaneously involves social (maternal separation and mixing with unfamiliar peers) and environmental stressors (handling, transport and housing in a novel pen). These stressors together often lead to a low feed (energy) intake (reviewed by^3^), gastro-intestinal problems (reviewed by^4^), gut microbiota dysbiosis (reviewed by^5^), reduced growth and behavioural disturbances^6,7^ after weaning, thereby reducing health and welfare. Stimulating the consumption of solid feed prior to weaning, i.e. creep feed, may improve piglet adaptation after weaning^8–11^, but factors influencing creep feed intake in piglets are unclear up to now.

Based on the findings by Algers *et al*.^12^, it was suggested that energy availability from milk affects individual creep feed intake. The proposed ‘compensatory feeding hypothesis’ suggests that slower-growing piglets in the litter will compensate for a lower energy intake from milk by consuming more creep feed and therefore undergo a less severe nutritional challenge at weaning and grow faster after weaning. Piglets classified as ‘good eaters’ of creep feed or with a long time (eating) at the feeder before and after weaning were, indeed, slower-growing piglets before weaning^12–15^. Complementary, other data suggest that the compensatory feeding hypothesis also holds on litter level, which may partly explain the large between-litter variation in creep feed intake^8,9^. Firstly, litters from intermittent suckling regimes that experienced reduced growth, due to separation from the sow for multiple hours per day, had a higher creep feed intake^16,17^, post-weaning feed intake^10,18^ and post-weaning weight gain^19^ than faster-growing conventional litters. However, effects of a low energy intake from milk are confounded in intermittent suckling regimes by the temporal maternal separation before weaning, which may increase separation stress before weaning, but reduce separation stress after weaning^20^. Secondly, primiparous sows, that produce less milk^21–23^ and consequently wean lighter piglets^14,24^, have been found to rear more eaters of creep feed than second parity sows, and particularly more moderate eaters of creep feed compared to multiparous sows^14^. Results of other studies, however, do not support or are even contradictory to the compensatory feeding hypothesis^25–27^.

It is likely that the consumption of creep feed is not solely driven by milk availability, as the milk production by the sow already becomes limiting for piglet growth from one week of lactation^28^ and a substantial proportion of piglets do not consume creep feed before they are weaned at 3 to 4 weeks of lactation^10,11,27^. Several studies suggested that creep feed intake may also be driven by intrinsic exploration in piglets towards the feed(er)^29–32^. Exploratory behaviour is closely related to play behaviour as both are likely involved in ‘object play’ in piglets, which includes holding or carrying an object or material in the mouth, manipulating, shaking and tugging it^33^. Object exploration is often hard to distinguish from and precedes object play (reviewed by^34^). In addition, exploratory behaviour, e.g. nosing, rooting, chewing, and object play seem to follow a similar developmental pattern in piglets. Both initiate in the first week of life and peak around four weeks of age, during which ingestion of solid feed items starts to increase^35,36^. It is therefore possible that exploratory behaviour and object play are involved in the development of feed intake behaviour in suckling piglets.

Our aim was to establish whether a low energy intake (homeostatic drive) from milk, by means of maternal feed restriction, and presenting creep feed in an exploratory and playful context (exploratory drive), by means of a foraging-stimulating play-feeder, are important and potentially interacting factors in getting piglets to eat creep feed. Subsequently, we studied the effects of the two factors on post-weaning adaptation in terms of behaviour and performance. We hypothesized that both factors would encourage piglets to familiarize themselves with creep feed and thereby reduce the stress from dietary changes at weaning and improve their adaptation after weaning.

## Methods

The protocol of the experiment (sows: AVD104002015325, piglets: AVD104002016515) was approved by the Animal Care and Use committee of Wageningen University & Research (Wageningen, The Netherlands) and in accordance with the Dutch law on animal experimentation, which complies with the European Directive 2010/63/EU on the protection of animals used for scientific purposes. The use of Indigo carmine as feed colorant was approved by the Medicines Evaluation Board (Utrecht, The Netherlands).

### Feeding strategies of sows and piglets during lactation

The study was set up as a 2 × 2 factorial arrangement with sow feeding (SF, lactational feed intake level) and piglet feeding (PF, creep feed presentation strategy) as experimental factors. All sows were fed the same level of commercially available sow diet twice a day (7:30 and 16:00 h) until d9 post-partum (8.9 MJ/kg as-fed net energy, 133 g crude protein, 5.6 standardized ileal digestible lysine/kg dry matter, ‘Standaard zeugenbrok’ during gestation and 9.3 MJ/kg as-fed net energy, 156 g crude protein, 7.7 standardized ileal digestible lysine/kg dry matter, ‘Zeugen Maxima Lacto’ during lactation, AgruniekRijnvallei, Wageningen, The Netherlands). The sows were provided 2.9 kg/d between d102–109 of gestation and the amount was gradually reduced to 2 kg/d at parturition, and increased thereafter with 0.5 kg/d. From d10 post-partum onwards, sows were fed in three feedings (7:00, 13:00 and 19:00 h) and received either 6.5 kg feed per day (full-fed, FF, n = 19) or 3.25 kg feed per day (restrictedly-fed, RES, n = 18) to create a contrast in energy supply from milk. The milk production of RES-sows was indeed lower compared to FF-sows, at least between d17–24 of lactation, and the milk of RES-sows contained a lower percentage of fat (Costermans *et al*., submitted). Moreover, RES-sows lost more body weight (19.2 ± 2.0 vs. 14.2 ± 2.0 kg) and muscle depth of the longissimus dorsi (1.3 ± 0.1 vs. 0.6 ± 0.1 mm) between d10–24 than FF-sows, but feed restriction of the sow did not affect back fat loss (3.4 ± 0.6 vs. 2.5 ± 0.6 mm)^37^. These changes were accompanied by lower plasma insulin-like growth factor 1 levels and higher plasma creatinine levels of RES-sows from d17 onwards, indicating a more severe negative energy balance of RES-sows^37^. A more detailed description of the effects of feed restriction during lactation on sow (reproductive) performance, energy mobilisation and milk quality are reported in Costermans *et al*.^37^ and Costermans *et al*. (submitted). Piglets reared by RES-sows will be named ‘RES-piglets’ and piglets reared by FF-sows will be named ‘FF-piglets’.

**Figure 1:**
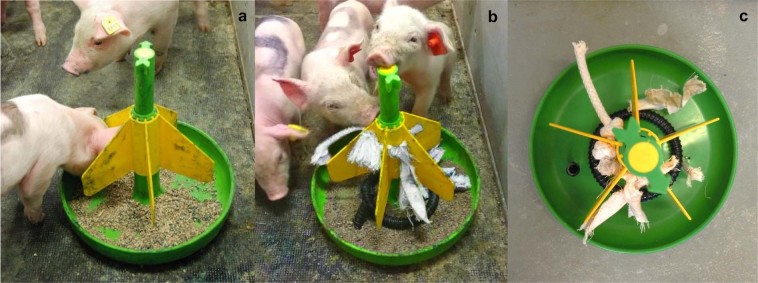
Litters were either creep-fed in a conventional feeder (**a**) or in a foraging-stimulating ‘play-feeder’ (**b, c**) from d4. The play-feeder was created by attaching canvas clothes, cotton ropes and PVC spiral tubes to the conventional feeder.

The litters were either creep-fed in a round conventional feeder (CON, n = 18 of which 9 FF and 9 RES; MS Clickfeeder Mini, MS Schippers, Bladel, The Netherlands) or in a foraging-stimulating ‘play-feeder’ (PL, n = 19 of which 10 FF and 9 RES) from d4 after birth. The piglet feeders had a diameter of 26 cm and five feeding places (Fig. 1a). The play-feeder was created by attaching canvas clothes (purchased at local market), braided natural cotton ropes (10 mm in diameter, MS Schippers, Bladel, The Netherlands) and PVC spiral tubes (13 mm in diameter, Ubbink, Alkmaar, The Netherlands) to the conventional feeder (Fig. 1b,c). These materials were attached on the inside at the bottom of the feeder (4 pieces of canvas clothes, 1 cotton rope, 1 PVC spiral tube). Two canvas clothes and one cotton rope were also tied to the feeder so they were hanging down and a PVC spiral ring was placed around the mounting system of the feeder so that it could be moved up and down. In this way the play-feeder allowed piglets to express object play as well as foraging behaviours, i.e. rooting, nosing, chewing, biting, pushing, pulling and lifting. A video showing interaction of piglets with the play-feeder can be found online as Supplementary Video S1. The canvas clothes and cotton ropes were replaced at d18. Feeders were located at the front end of the farrowing area near the feeding corridor.

Piglets were fed an experimental creep feed as crumble (Research Diet Services, Wijk bij Duurstede, The Netherlands). The creep feed (11.8 MJ/kg as-fed net energy, 195 g crude protein, 11.9 g standardized ileal digestible lysine/kg dry matter) was high in dietary non-starch polysaccharides (261 g/kg dry matter), originating from cereal grains, sugarbeet pulp, oat hulls, galacto-oligosaccharides, inulin and high-amylose starch (Supplementary Tables S2 and S3). Feed colorant Indigo carmine (E132 Eurocert 311811, Sensient Food Colors, Elburg, The Netherlands) was added manually to the pre-weaning piglet diet (5 g/kg of feed) to allow for identification of eaters (see below). Every morning a small scoop of fresh creep feed was given and feeders were checked at least twice daily and three times a day from d10 to provide the creep feed *ad libitum*. To maintain freshness of the creep feed, all feed was replaced and feed bowls were cleaned using paper towels and water at each feed weigh-back (see below). Two days prior to weaning, the creep feed was mixed with a commercially available crumbled nursery diet to familiarize piglets to the nursery diet before weaning (161 g crude protein, 48 g crude fibre and 11.8 g standardized ileal digestible lysine/kg dry matter, ‘Insecto Speen’, Coppens Diervoeding, Helmond, The Netherlands).

Litter size was standardized by cross-fostering within 3 days of age, resulting in an average number of 12.7 ± 0.2 piglets per litter at d4. The thirty-seven sows and their litters were allotted to one of four treatments (piglet feeding at d4 after birth and sow feeding at d10 post-partum) based on sow body weight and P2 back fat thickness at parturition and piglet birthdate and body weight at d0 and d4. Litter size at weaning did not differ between treatments (on average 12.4 ± 0.2 piglets per litter at d24).

### Animals, housing and management

Pregnant primiparous TN70 sows (Norwegian Landrace x Large White, Topigs Norsvin, Vught, The Netherlands), inseminated with Tempo boar semen, were divided over two farrowing rooms (balanced for treatments) in three consecutive batches. The sows originated from one conventional farm and were housed at research facility Carus (Wageningen University & Research, The Netherlands) from two weeks before farrowing onwards. The pen was equipped with a farrowing area (2.85 × 1.80 m) and a free-movement area (1.85 × 1.80 m). The farrowing area was equipped with a crate (2.85 × 0.60 m) including a feed trough, a drinking nipple, a jute sack (around farrowing), a long-stemmed straw dispenser (from d10 post-partum) and three chew objects that alternated two times a week (metal chain with either one of three attachments: bolts, ball or PVC pipe) for the sow. The farrowing area included a drinking nipple, chew object (metal chain with bolts) and heating lamp for the piglets. The floor consisted of slats and rubber mats (2.00 × 1.75 m, in farrowing area) that served as nest for the piglets and provided lying comfort to the sow. The free-movement area was equipped with a drinking nipple for the sow. The sows were loose housed, except from shortly before farrowing until the first four days post-partum to minimize piglet crushing. During this initial period the free-movement area was not accessible to sows and piglets. Within 24 hours after birth, piglets received an ear tag and intramuscular iron injection and their birth weight and sex were determined. No teeth clipping, tail docking or castration were performed. In the farrowing rooms lights and radio were on from 6:00 to 19:30 h and lights were dimmed during the night. The room temperature was 23 °C around farrowing and gradually decreased to 20 °C at weaning.

At weaning (24.3 ± 0.1 days of age) a subset of 144 piglets (n = 9 weaner pens per treatment) was relocated in two weaner rooms (balanced for treatments) in two batches. Piglets were mixed with conspecifics from the same pre-weaning treatment and housed in pens (2.76 × 1.20 m) with four piglets (two males and two females) from three litters. Piglets were selected based on their sex and their body weight at d22 (close to the average weight of the litter and treatment group). Piglets with a history of medication and leg/claw problems were excluded from selection. The weaning weight of the selected piglets was 5.82 ± 0.13 kg, 6.06 ± 0.05 kg, 6.66 ± 0.04 kg and 6.30 ± 0.06 kg for RES-CON, RES-PL, FF-CON and FF-PL respectively. All weaner pens were identical and equipped with a conventional feed trough (three feeding spaces), drinking trough and chew object (metal chain with bolts). The floor was party slatted and partly covered with a rubber mat (1.75 × 1.20 m) that provided lying comfort to the piglets and prevented spillage of feed through the slats. Piglets were fed the nursery diet *ad libitum*. In the weaner rooms lights and radio were on from 7:00 to 19:00 h and room temperature gradually decreased from 25 to 23 °C at two weeks post-weaning, when the experiment ended and piglets were 39 days of age.

### Measurements

#### Piglet growth

Piglets were individually weighed at d0 (within 24 h after birth), 4 (before commencing piglet feeding), 10 (before commencing feeding strategy of the sow), 17, 22, 24 (at weaning) before weaning and d1, 2, 5 and 15 post-weaning (at end of experiment).

#### Piglet behaviours

Piglets were marked using dark permanent hair dye (pre-weaning, Syoss professional performance permanent coloration, 1-1 noir) and animal marking spray (post-weaning, MS marking spray, MS Schippers, Bladel, The Netherlands). Feed-related behaviours in the farrowing pens were observed live at d9, 16 and 23 by 2-min instantaneous scan sampling for six sessions of one hour per day (i.e. 180 scans/piglet/d) using pen and paper. Sniffing, touching with snout or rooting the feed were defined as ‘exploring feed’ and eating or chewing feed was defined as ‘eating feed’. Sniffing, touching with snout, rooting or chewing on (toys of) the feeder, pushing or lifting toys of the feeder or shaking head while having toy of feeder in mouth were classified as ‘exploring feeder’. Feeder exploration and object play with (toys of the) the feeder are grouped as one behaviour in our study, as object exploration is often hard to distinguish from and precedes object play (reviewed by^34^). ‘Suckling’ was recorded on litter level as drinking milk from teat of sow (soft suckling noises). Observations were used to calculate time spent on feed-related behaviours, the proportion of piglets visiting the feeder (piglets that were scored either exploring the feed, exploring the feeder or eating feed at least once) and the proportion of piglets eating (piglets scored eating feed at least once). Behaviours in the weaner pens were recorded live on 30 and 39 days of age (6 and 14 days post-weaning respectively) by 2-min instantaneous scan sampling for six one-hour sessions per day using a Psion hand-held computer with the Pocket Observer 3.1 software package (Noldus Information Technology, Wageningen, The Netherlands). The ethogram with behaviours of interest in the weaner rooms is listed in Supplementary Table S4. Observation sessions started at 8:00, 9:15 and 10:30 h in the morning and at 14:00, 15:15 and 16:30 h in the afternoon.

#### Creep feed eaters and feed intake

Rectal swabs were taken at d10, 17, 22 and 24 to assess the presence of blue colour (Indigo carmine) and thereby to determine qualitative intake of creep feed (yes or no) on piglet level. Blue colour was not detected on the swabs at d10 and therefore this time point was excluded from analyses. Piglets were subsequently classified into different eater classes after Collins *et al*.^38^. Piglets of which blue colour was present on the swab on three measurement days were classified as ‘good/early eaters’. ‘Moderate eaters’ had blue colour on the swab on two measurement days and ‘bad eaters’ on only one measurement day. This may, by definition, not necessarily concern the last measurement day(s) and therefore these piglets cannot be called ‘late eaters’. Swabs of ‘non-eaters’ did not include blue colour at any of the measurement days. Pre-weaning feed intake was determined per litter between d10-17, d17-22 and d22-24 and post-weaning feed intake was determined per pen between 0-4h , 4-24h, d1-2, d2-5 and d5-15 post-weaning.

#### Body lesions and damage on piglets

We monitored the number of body lesions on the piglets at 4 hours and d1, d2 and d15 post-weaning (as a measure of aggression, according to Turner *et al*.^39^) and classified bite injuries on ears and tails of the piglets into no damage, bite marks or small wound at d15 post-weaning (as a measure of oral manipulation, according to van Nieuwamerongen *et al*.^40^).

#### Faecal consistency scores of piglets

Faeces in the weaner pens were scored daily for consistency according to Pedersen and Toft^41^. Score 1 (firm and shaped) and 2 (soft and shaped) represent normal faeces, and were combined into one score prior to data analysis. Score 3 (loose) and 4 (watery) represent diarrhoea. The highest faecal consistency score that was observed in a pen was selected on each measurement day and averaged over two weeks post-weaning to calculate the mean faecal consistency score (FCS) per pen. Faeces were removed on a daily basis after scoring to guarantee consistency scoring of fresh faeces.

### Statistical analyses

#### Data processing

Piglet behaviours in the home pen were averaged per piglet per day and expressed as proportions of time. Eating feed was very low at d9 and exploring feed was very low at all ages before weaning (<0.1% of observation time) and were therefore excluded from analysis. After weaning, exploring feed, feeder and drinking trough were pooled into ‘exploring feed(er) and drinker’. Nosing, rooting and chewing the environment as well as chewing air were combined into ‘exploring environment’. The behavioural element ‘chewing poo’ was excluded from analyses because it was seen very rarely (0.01% of observation time). Lying with eyes closed and lying with eyes open were pooled into ‘inactive behaviour’. Standing and walking were pooled into ‘standing and walking’. Playing individually, socially and with chew object were summed and presented as ‘playing’. To investigate ‘nosing pen mates’, nosing body and snout contact were merged. The ear of the piglets with the highest damage score (either the left or right ear) was used in the analysis of ear damage. The number of body lesions on the piglets at weaning was subtracted from the number of body lesions at 4 hours after weaning.

#### Data analyses

Data were analysed with mixed models in the statistical software SAS 9.4 (SAS Institute Inc., Cary, NC, USA). Proportions of time spent on the different behaviours, the percentage of piglets per litter visiting the feeder and eating, the percentage of eaters per litter identified by rectal swab, and uniformity in BW (expressed as coefficient of variation) were analysed using a generalised linear mixed model (GLIMMIX) with a binomial distribution, logit link function and an additional multiplicative overdispersion parameter. The model did not converge with nosing environment at 2 weeks post-weaning as response variable, therefore this variable was analysed in a linear mixed model (MIXED). The number of body lesions and the number days with post-weaning diarrhoea were analysed using a GLIMMIX with a Poisson distribution, log link function and an additional multiplicative overdispersion parameter. The model did not converge with the total number of body lesions at 2 weeks post-weaning as response variable and was therefore analysed in a MIXED after square root transformation. Tail damage of weaner piglets was analysed in a GLIMMIX with a multinomial distribution and a cumulative logit link function. Data on the prevalence of watery diarrhoea in weaner pens were expressed as binary data and analysed with a GLIMMIX with a logit link and binary distribution. Individual creep feed classification of suckling piglets and ear damage on weaner piglets were analysed in a Fisher’s exact test, because there was an empty sub-classification category for the interaction effects. Continuous variables creep feed intake, average daily feed intake (ADFI), average daily gain (ADG), body weight (BW), feed conversion ratio (FCR) and mean faecal consistency score (FCS) were analysed in a MIXED procedure. For feed intake and ADG, totals over the pre- (d10–24) and post-weaning period (d0–15 post-weaning) were analysed, as well as effects on separate periods. Model residuals of the MIXED procedure were checked for normality. If model residuals were not normally distributed, data were transformed before analyses.

The model included the fixed effects of sow feeding (RES vs. FF), piglet feeding (PL vs. CON), their interactions, as well as batch (batch 1, 2 or 3). In the analysis of tail damage on weaner piglets, batch was excluded from the multinomial model (not significant). The number of body lesions at 4, 24 and 48 h post-weaning were analysed in a repeated GLIMMIX, therefore also including day and its interactions with the treatment groups as fixed effect. The number of body lesions at 2 weeks post-weaning was analysed separately in a GLIMMIX. Post-weaning ADFI and ADG were analysed in a repeated GLIMMIX and the four periods were also analysed separately. The same results were obtained and the results of the separate analyses are reported to facilitate interpretation. In addition, for behaviour, BW, ADG and the number of body lesions, the model included a random pen effect, nested within treatments and batch (farrowing pen for pre-weaning measurements and weaner pen for post-weaning measurements). Significant fixed effects were further analysed using differences of least squares means. Correlations were calculated at litter level using Pearson’s (normally distributed variables) and Spearman’s correlation coefficients (not normally distributed variables). Data are presented as (untransformed) means ± SEM based on pen averages. Differences at *P* < 0.05 were considered statistically significant and differences at 0.05 ≤ *P* < 0.10 were considered a trend.

## Results

### Feed-related behaviour and percentage of eaters before weaning

**Figure 2:**
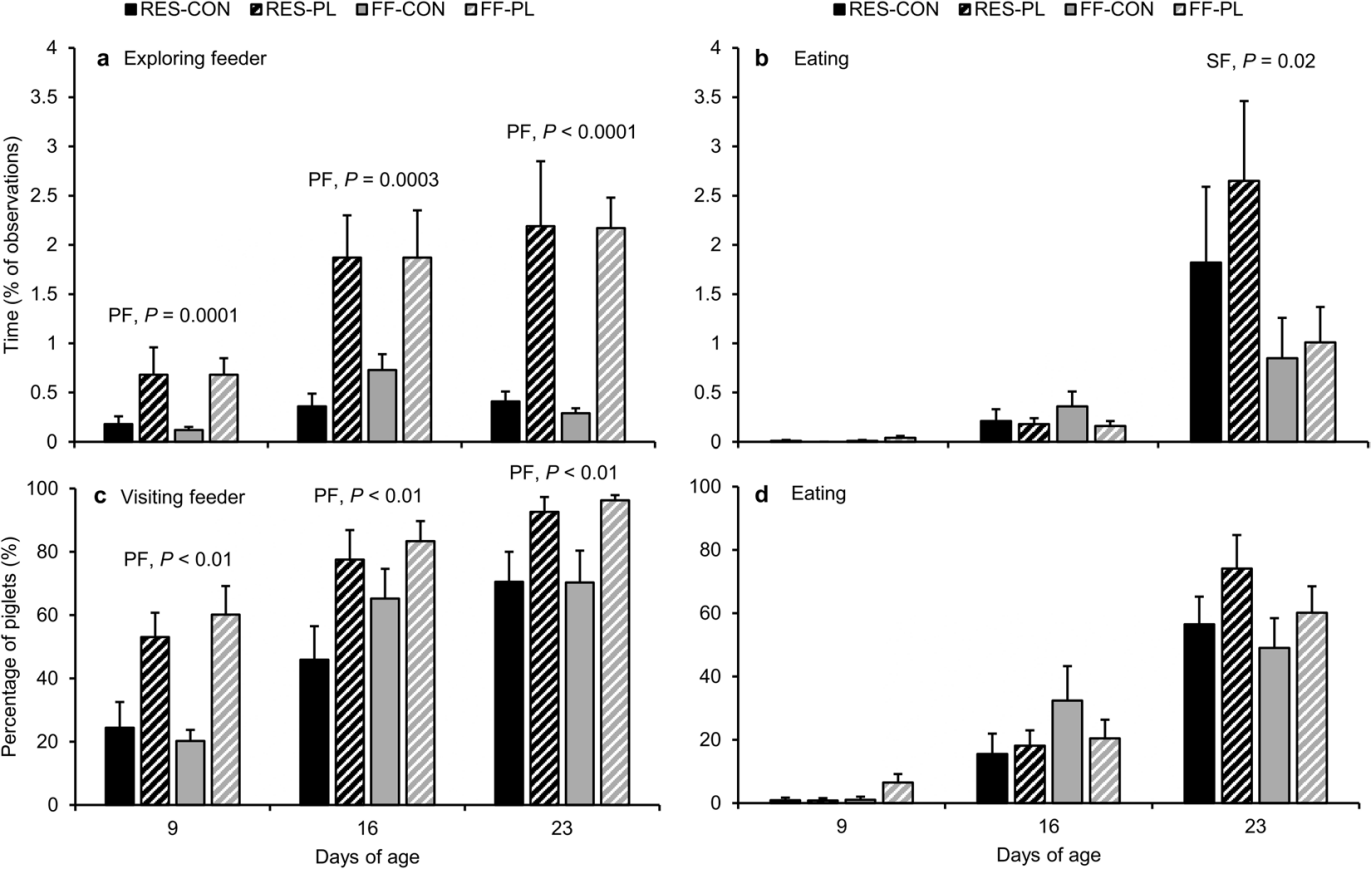
Time spent exploring the feeder (**a**) and eating (**b**) and the percentage of piglets per litter visiting the feeder (**c**) and eating (**d**) based on live home pen observations. Piglets were reared by restrictedly-fed sows (RES) or full-fed sows (FF) and their creep feed presented in a control feeder (CON) or play-feeder (PL) prior to weaning. SF = sow feeding. PF = piglet feeding. Data are expressed as means ± SEM based on pen averages and were analysed with a generalised linear mixed model.

The play-feeder increased the time spent exploring the feeder by 3.5 to 6 times, irrespective of feed intake of the sow (PL vs. CON, d9: 0.68 ± 0.16 vs. 0.15 ± 0.04%; d16: 1.87 ± 0.32 vs. 0.54 ± 0.11%; d23: 2.18 ± 0.34 vs. 0.35 ± 0.05%; Fig. 2a). The treatments did not affect time spent eating creep feed at d16 (Fig. 2b), however RES-piglets doubled their time spent eating compared to FF-piglets at d23 (RES: 2.23 ± 0.55 vs. FF: 0.94 ± 0.26%). Time spent exploring the feeder correlated with time spent eating (r = 0.57; *P* < 0.0001) at all observation days (d9: r = 0.46, *P* < 0.01; d16: r = 0.51, *P* = 0.001; d23: r = 0.37, *P* = 0.02). Irrespective of feed intake of the sow and creep feed presentation method, the percentage of piglets within the litter visiting the feeder was 40, 68 and 83% at d9, 16 and 23 and the percentage observed to be eating was 2, 22 and 60% respectively. The play-feeder stimulated more piglets within a litter to visit the feeder (PL vs. CON, d9: 56.8 ± 5.9 vs. 22.3 ± 4.3%; d16: 80.6 ± 5.4 vs. 55.6 ± 7.3%; d23: 94.5 ± 2.4 vs. 70.4 ± 6.7%; Fig. 2c), but not to eat (Fig. 2d).

**Figure 3:**
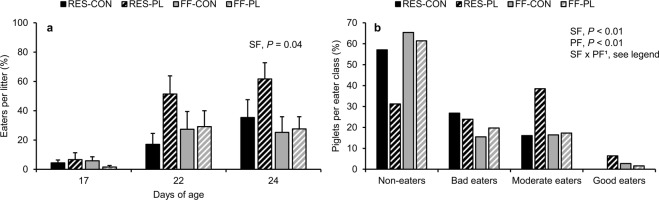
The percentage of eaters per litter over time (**a**) and individual creep feed classification of piglets (**b**) based on the presence of blue colour on rectal swabs. Piglets were reared by restrictedly-fed sows (RES) or full-fed sows (FF) and their creep feed presented in a control feeder (CON) or play-feeder (PL) prior to weaning. SF = sow feeding. PF = piglet feeding. Data are expressed as means ± SEM based on pen averages and were analysed with a generalised linear mixed model (percentage of eaters) and a Fisher’s exact test (creep feed classification). ^1^SF effect within PL: *P* < 0.0001 and within CON: *P* = 0.07. PF effect within RES: *P* < 0.0001 and no effect within FF: *P* = 0.78.

The percentage of creep feed eaters per litter, as identified by blue coloured swabs, was generally low up to d17, but increased with age (d10: 0%, d17: 5%, d22: 31%, d24: 37%). SF, PF and their interaction did not affect the percentage of eaters at d17 and 22. At weaning, litters of RES-sows included double as much eaters than litters of FF-sows (48.6 ± 8.6 vs. 26.5 ± 6.5% eaters/litter; Fig. 3a). The number of eaters per litter based on observations (seen eating at least once per observation day) correlated with the number of eaters per litter determined by the rectal swabs (blue colour on the swab) that were taken the day after (r = 0.71, *P* < 0.0001). Maternal feed restriction stimulated the number of piglets in better eater classes (*P* < 0.01) and the effect was more pronounced in litters that had access to a play-feeder, as shown in Fig. 3b (SF effect within PL: *P* < 0.0001, particularly moderate and good eaters, and SF effect within CON: *P* = 0.07, particularly bad eaters). The play-feeder also increased the number of piglets in better eater classes (*P* < 0.01, particularly the proportion of moderate and good eaters), but only within RES-piglets (PF effect within RES: *P* < 0.0001 and no effect within FF: *P* = 0.78).

### Creep feed intake

Creep feed intake was generally low (especially up to d17 with on average ≤8 g/piglet) with on average 62 g/piglet between d4–24, and variable between litters, with total intake ranging from 3 to 399 g/piglet. As feed intake could be measured on litter level only, this was calculated as the intake per litter divided by the number of piglets in the litter and therefore the feed intake of individual piglets could be higher than 399 g. About 50% of the total feed intake was consumed during the last two days prior to weaning. Treatments did not affect feed intake between d17–22, but RES-piglets consumed more feed the last two days before weaning than FF-piglets (49 ± 13.9 vs. 15 ± 4.3 g/piglet; Table 1). Overall from the start of maternal feed restriction until weaning, RES-piglets tended to eat double the amount of feed compared to FF-piglets (d10–24: 81 ± 22.6 vs. 41 ± 10.7 g/piglet). Time spent exploring the feeder correlated with feed intake (r = 0.46; *P* < 0.0001). Analyses per day showed that time spent exploring the feeder did correlate with feed intake at d9 (r = 0.45, *P* < 0.01) and d16 (r = 0.31, *P* = 0.05), but not at d23 (r = 0.07, *P* = 0.66). Furthermore, time spent eating feed strongly correlated with feed intake (r = 0.83, *P* < 0.0001).

**Table 1:** Pre-weaning performance of piglets reared by restrictedly-fed sows (RES) or full-fed sows (FF) and their creep feed presented in a control feeder (CON) or play-feeder (PL) prior to weaning.

	RES		FF		Significance		
	CON	PL	CON	PL	SF	PF	SF × PF
**Creep feed intake, g/piglet**							
d 17–22	18.0 ± 5.2	35.8 ± 17.1	23.8 ± 11.4	17.9 ± 6.8	0.44	0.54	0.52
d 22–24	38.9 ± 13.8	59.4 ± 24.7	13.7 ± 4.5	16.4 ± 7.3	**0.01**	0.74	0.80
d 10–24^a^	61.5 ± 18.1	101.1 ± 41.8	44.6 ± 17.5	38.4 ± 13.8	**0.09**	0.58	0.66
**ADG, g/piglet/d**							
d 10–17	145 ± 7	149 ± 8	192 ± 7	185 ± 8	**<0.0001**	0.97	0.35
d 17–24	205 ± 8	212 ± 9	263 ± 10	260 ± 11	**<0.0001**	0.74	0.50
d 10–24	208 ± 9	219 ± 9	259 ± 9	249 ± 10	**<0.0001**	0.87	0.19
**Body weight, kg**							
d 0	1.37 ± 0.06	1.41 ± 0.06	1.36 ± 0.04	1.41 ± 0.08	0.95	0.51	0.81
d 4	1.80 ± 0.12	1.87 ± 0.08	1.80 ± 0.12	1.91 ± 0.11	0.55	0.90	0.78
d 24	5.70 ± 0.23	6.15 ± 0.19	6.91 ± 0.42	6.54 ± 0.32	**0.01**	0.87	0.21
**Litter CV in BW, %**							
d 4	14.6 ± 1.3	15.5 ± 2.1	15.0 ± 1.4	14.6 ± 0.8	0.99	0.65	0.92
d 24	15.7 ± 1.8	17.1 ± 1.7	14.9 ± 1.9	16.4 ± 1.5	0.63	0.42	0.93

SF = sow feeding. PF = piglet feeding. Data are expressed as means ± SEM based on pen averages and were analysed with mixed models.

^a^Creep feed intake was low from d 10–17 and therefore not analysed separately.

### Piglet growth, body weight and litter uniformity in body weight before weaning

RES-piglets gained less weight between d10–17 (147 ± 5.3 vs. 188 ± 5.3 g/d), between d17–24 (209 ± 6.0 vs. 262 ± 7.4 g/d) and overall between d10–24 (213 ± 6.3 vs. 254 ± 6.7 g/d) than FF-piglets (Table 1). Consequently, RES-piglets tended to weigh less at d17 (4.47 ± 0.14 vs. 4.89 ± 0.22 kg; *P* = 0.09) and weighed less at weaning compared to FF-piglets (5.92 ± 0.16 vs. 6.72 ± 0.26 kg; Table 1). PL-piglets did not differ in ADG during lactation or BW at weaning from CON-piglets. SF, PF and their interaction did not affect homogeneity in BW within litters (CV) from d4 to weaning (Table 1).

### Piglet behaviour after weaning

#### Ingestive behaviour

PL-piglets spent more time eating at week 2 after weaning compared to CON-piglets (PL: 11.8 ± 0.5 vs. CON: 10.4 ± 0.4%; Table 2). Moreover, PL-piglets showed more drinking than CON-piglets at week 1 (PL: 1.0 ± 0.1 vs. CON: 0.7 ± 0.1%) and week 2 post-weaning (PL: 1.2 ± 0.1 vs. CON: 0.9 ± 0.1%). Drinking was lower for RES-piglets compared to FF-piglets at week 2 post-weaning (RES: 0.8 ± 0.1 vs. FF: 1.2 ± 0.1%).

**Table 2:** Behavioural activities (% of total observations) in the first two weeks after weaning (week 1: 30 days of age, week 2: 38 days of age) of piglets reared by restrictedly-fed sows (RES) or full-fed sows (FF) and their creep feed presented in a control feeder (CON) or play-feeder (PL) prior to weaning.

Behaviour	RES		FF		Significance		
	CON	PL	CON	PL	SF	PF	SF × PF
*Week 1 after weaning*							
**‘Ingestive behaviour’**							
Eating feed	13.2 ± 0.7	13.1 ± 0.7	14.2 ± 1.2	13.5 ± 0.9	0.42	0.68	0.74
Drinking	0.7 ± 0.1	0.9 ± 0.1	0.6 ± 0.1	1.2 ± 0.2	0.61	**0.02**	0.54
**‘Exploratory behaviour’**							
Exploring feed(er) and drinker	2.7 ± 0.3	2.9 ± 0.5	1.9 ± 0.4	2.0 ± 0.3	**0.03**	0.70	0.99
Exploring environment	22.5 ± 2.1	25.6 ± 2.2	18.2 ± 2.4	18.1 ± 1.0	**0.01**	0.43	0.59
**‘Postures and locomotion’**							
Inactive behaviour	45.0 ± 3.5	40.7 ± 2.9	50.2 ± 4.2	47.2 ± 1.9	**0.08**	0.27	0.84
Standing and walking	6.6 ± 0.6	6.0 ± 0.6	5.9 ± 0.7	7.1 ± 0.8	0.84	0.73	0.21
**‘Play behaviour’**							
Playing	2.1 ± 0.3	2.3 ± 0.4	2.2 ± 0.4	2.8 ± 0.5	0.68	0.37	0.59
**‘Pig-directed behaviour’**							
Nosing pen mates	1.4 ± 0.2	1.8 ± 0.3	1.7 ± 0.4	1.7 ± 0.2	0.75	0.36	0.69
Ear biting	0.3 ± 0.1	0.4 ± 0.1	0.4 ± 0.1	0.4 ± 0.1	0.68	0.48	0.38
Tail biting	0.4 ± 0.1	0.3 ± 0.1	0.3 ± 0.1	0.3 ± 0.1	0.85	0.85	0.68
Belly nosing	0.1 ± 0.03	0.5 ± 0.3	0.4 ± 0.2	0.5 ± 0.2	0.16	**<0.10**	0.14
Manipulating pen mates	1.9 ± 0.3	2.2 ± 0.3	1.8 ± 0.4	2.0 ± 0.5	0.53	0.55	0.96
Mounting pen mates	1.2 ± 0.2	1.2 ± 0.3	0.9 ± 0.2	1.2 ± 0.1	0.49	0.52	0.61
Aggression	0.8 ± 0.2^a^	0.8 ± 0.2^a^	0.3 ± 0.1^b^	0.8 ± 0.1^a^	0.11	**0.04**	**0.04**
*Week 2 after weaning*							
**‘Ingestive behaviour’**							
Eating feed	10.5 ± 0.6	11.4 ± 0.7	10.4 ± 0.5	12.2 ± 0.8	0.57	**0.01**	0.40
Drinking	0.8 ± 0.1	0.9 ± 0.2	1.0 ± 0.1	1.5 ± 0.2	**0.03**	**0.03**	0.49
**‘Exploratory behaviour’**							
Exploring feed(er) and drinker	2.0 ± 0.3	2.3 ± 0.1	2.3 ± 0.2	3.0 ± 0.2	**0.06**	**0.05**	0.45
Exploring environment	26.0 ± 1.7	25.9 ± 1.9	27.2 ± 1.9	21.7 ± 2.2	0.39	0.13	0.14
**‘Postures and locomotion’**							
Inactive behaviour	46.0 ± 1.7	45.6 ± 2.4	44.0 ± 3.0	45.7 ± 3.9	0.72	0.86	0.76
Standing and walking	4.7 ± 0.4	4.0 ± 0.6	4.5 ± 0.4	5.0 ± 0.6	0.42	0.75	0.26
**‘Play behaviour’**							
Playing	1.8 ± 0.2	1.8 ± 0.2	2.1 ± 0.4	2.4 ± 0.4	0.33	0.53	0.44
**‘Pig-directed behaviour’**							
Nosing pen mates	1.9 ± 0.3	1.3 ± 0.2	1.5 ± 0.2	1.6 ± 0.2	0.84	0.33	0.16
Ear biting	0.4 ± 0.1	0.3 ± 0.1	0.8 ± 0.2	0.6 ± 0.1	**<0.01**	0.23	0.93
Tail biting	0.5 ± 0.1	0.5 ± 0.1	0.5 ± 0.1	0.4 ± 0.1	0.93	0.82	0.96
Belly nosing	0.6 ± 0.4	0.4 ± 0.2	0.4 ± 0.2	0.7 ± 0.3	0.66	0.78	0.52
Manipulating pen mates	2.3 ± 0.4	2.7 ± 0.3	2.6 ± 0.4	2.1 ± 0.3	0.67	0.98	0.19
Mounting pen mates	0.6 ± 0.1	0.6 ± 0.1	0.8 ± 0.2	0.8 ± 0.2	0.46	1.00	1.00
Aggression	0.8 ± 0.2	1.0 ± 0.2	1.0 ± 0.1	0.9 ± 0.2	0.88	0.79	0.53

SF = sow feeding. PF = piglet feeding. Data are means ± SEM based on pen averages and were analysed with generalised linear mixed models. Within a row superscripts without a common letter differ at *P* < 0.05.

#### Exploratory behaviour

RES-piglets had a higher level of exploration towards the feed(er) and drinker compared to FF-piglets at week 1 post-weaning (RES: 2.8 ± 0.3 vs. FF: 2.0 ± 0.2%), but tended to have a lower level of exploration towards the feed(er) and drinker at week 2 post-weaning (RES: 2.1 ± 0.2 vs. FF: 2.6 ± 0.2%). Moreover, RES-piglets spent more time on exploring the environment at week 1 post-weaning compared to FF-piglets (RES: 24.1 ± 1.5 vs. FF: 18.1 ± 1.2%). PL-piglets tended to explore the feed(er) and drinker more at week 2 post-weaning in comparison with CON-piglets (PL: 2.6 ± 0.1 vs. CON: 2.1 ± 0.2%).

#### Postures and locomotion

RES-piglets tended to show less inactive behaviour compared to FF-piglets at week 1 post-weaning (RES: 42.9 ± 2.3 vs. FF: 48.7 ± 2.3%). The play-feeder did not affect (in)active behaviour after weaning.

#### Play behaviour

Playing was not affected by treatments.

#### Pig-directed behaviour

Ear biting was lower for RES-piglets compared to FF-piglets at week 2 post-weaning (RES: 0.4 ± 0.05 vs. FF: 0.7 ± 0.10%). PL-piglets tended to have higher levels of belly nosing compared to CON-piglets at week 1 post-weaning (PL: 0.5 ± 0.2 vs. CON: 0.2 ± 0.1%). The interaction between SF and PF affected aggression at week 1 post-weaning (*P* = 0.04), with less time spent on aggression in FF-CON piglets compared to FF-PL (*P* < 0.01), RES-CON (*P* < 0.05) and RES-PL piglets (*P* < 0.05). No effects were found on nosing pen mates, manipulating pen mates, tail biting and mounting.

#### Other behaviour

No interaction and main effects of SF and PF were found for the other behaviours after weaning (comfort behaviour: 0.42 ± 0.03%; eliminating: 0.68 ± 0.03%; data not shown).

### Body lesions and damage on piglets after weaning

#### Body lesions in the first two days post-weaning

The number of fresh body lesions was high the first day after weaning, but decreased thereafter with 16.8 ± 1.9, 22.2 ± 2.6 and 11.0 ± 1.8 lesions at 4, 24 and 48 hours post-weaning respectively. Interactions between SF x PF were found on the number of body lesions throughout the first two days after weaning (*P* < 0.02; no interaction with time). RES-piglets had less body lesions than FF-piglets, but only within CON (*P* < 0.01; RES-CON: 10.6 ± 2.2 vs. FF-CON: 23.7 ± 4.8 lesions, with intermediate levels of RES-PL: 10.8 ± 1.8 and FF-PL: 11.1 ± 1.8 lesions).

#### Body lesions and damage at week 2 post-weaning

**Figure 4:**
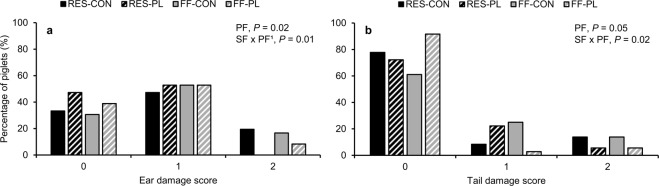
Occurrence of ear (**a**) and tail damage (**b**) (% of piglets with each score) at two weeks post-weaning on piglets reared by restrictedly-fed sows (RES) or full-fed sows (FF) and their creep feed presented in a control feeder (CON) or play-feeder (PL) prior to weaning. SF = sow feeding. PF = piglet feeding. 0: no damage, 1: small bite marks with the size of a pinhead, 2: small wound. Data are expressed as means and were analysed in a Fisher’s exact test (ear damage) and generalized linear mixed model with multinomial distribution and cumulative link function (tail damage). ^1^Play-feeder effect within FF.

On average 4.0 ± 0.4 lesions were found on the body of weaned piglets at two weeks after weaning. PL-piglets had a lower number of body lesions (3.1 ± 0.5 vs. 4.8 ± 0.6 lesions; Table 3). Moreover, there were less PL-piglets with wounds on their ears than CON-piglets (*P* = 0.02), but only when reared by FF-sows (PF effect within FF: *P* = 0.01; Fig. 4a). The percentage of piglets with tail damage was affected by the interaction between SF × PF (*P* = 0.02) and tended to be affected by the play-feeder (*P* = 0.05; Fig. 4b). RES-PL had less piglets with tail damage than RES-CON (*P* < 0.01, OR = 0.15, 95% CI = 0.04–0.61) and tended to have less piglets with tail damage than FF-PL (*P* = 0.06, OR = 0.26, 95% CI = 0.06–1.05). FF-CON and RES-CON (*P* = 0.20, OR = 1.96, 95% CI = 0.70–5.50) as well as FF-CON and FF-PL (*P* = 0.78, OR = 1.17, 95% CI = 0.40–3.44) did not differ in the proportion of piglets with tail damage.

**Table 3:** Post-weaning performance (d0–15 post-weaning) of piglets reared by restrictedly-fed sows (RES) or full-fed sows (FF) and their creep feed presented in a control feeder (CON) or play-feeder (PL) prior to weaning.

Variable	RES		FF		Significance		
	CON	PL	CON	PL	SF	PF	SF × PF
ADFI, d 0–15, g/piglet/d	311 ± 12	358 ± 15	288 ± 20	332 ± 16	0.12	**<0.01**	0.93
ADG, d 0–15, g/piglet/d	261 ± 10	305 ± 11	246 ± 11	281 ± 12	**0.06**	**<0.001**	0.61
FCR, d 0–15	1.20 ± 0.05	1.17 ± 0.01	1.16 ± 0.04	1.19 ± 0.04	0.75	0.95	0.46
BW at d 15, kg	9.73 ± 0.22	10.63 ± 0.13	10.35 ± 0.19	10.51 ± 0.23	0.27	**0.02**	0.11
Diarrhoea, days/pen	2.78 ± 0.72	2.00 ± 0.65	3.22 ± 0.64	1.44 ± 0.38	0.74	**0.04**	0.37
% of pens with ≥1 day watery diarrhoea	44.4	22.2	77.8	11.1	0.69	**0.01**	0.18
Faecal consistency score	0.23 ± 0.06	0.16 ± 0.06	0.28 ± 0.05	0.10 ± 0.03	0.94	**0.02**	0.28
Body lesions at d 15	4.7 ± 1.2	3.0 ± 0.7	4.8 ± 0.5	3.3 ± 0.8	0.35	**0.02**	0.91

SF = sow feeding. PF = piglet feeding. Data are expressed as means ± SEM based on pen averages and were analysed with mixed models. Within a row superscripts without a common letter differ at *P* < 0.01.

### Feed intake, growth and faecal consistency after weaning

**Figure 5:**
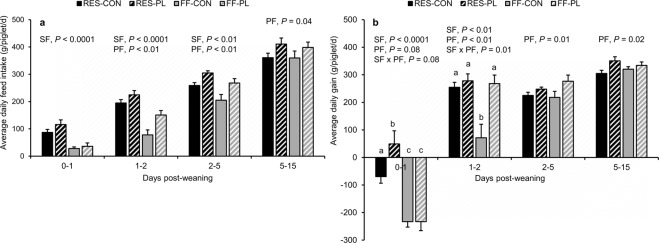
Average daily feed intake (**a**) and average daily gain (**b**) of weaner piglets reared by restrictedly-fed sows (RES) or full-fed sows (FF) and their creep feed presented in a control feeder (CON) or play-feeder (PL) prior to weaning. SF = sow feeding. PF = piglet feeding. Data are expressed as means ± SEM based on pen averages and were analysed with linear mixed models. Within a period superscripts without a common letter differ at *P* < 0.05.

Maternal feed restriction and the play-feeder increased feed intake in the first four hours after weaning (RES: 24 ± 4 vs. FF: 17 ± 3 g/piglet, PL: 24 ± 4 vs. CON: 17 ± 3 g/piglet; *P* < 0.05 for both). The treatments tended to interact in their effect on feed intake in this period (*P* = 0.08), showing that RES-PL had a higher intake of feed within the first four hours after weaning compared to the other three treatment groups (RES-CON: 17 ± 3, RES-PL: 31 ± 6, FF-CON: 16 ± 4, FF-PL: 18 ± 4 g/piglet; *P* ≤ 0.01 for comparisons). Maternal feed restriction and the play-feeder during lactation increased the ADFI (Fig. 5a) and ADG (Fig. 5b) after weaning, with the effect of the play-feeder lasting until the end of the experiment. Maternal feed restriction improved the intake of feed on the first day (RES: 101 ± 11 vs. FF: 32 ± 7 g/day), the second day (RES: 210 ± 10 vs. FF: 114 ± 15 g/day) and between d2–5 post-weaning (RES: 282 ± 9 vs. FF: 237 ± 15 g/day; Fig. 5a). The play-feeder improved the daily intake of feed on the second day (PL: 188 ± 14 vs. CON: 136 ± 18 g/day), between d2–5 (PL: 286 ± 10 vs. CON: 232 ± 13 g/day), between d5–15 (PL: 405 ± 14 vs. CON: 360 ± 15 g/day) and during the two weeks after weaning (PL: 345 ± 11 vs. CON: 300 ± 12 g/day; Table 3).

Interactions between SF × PF were found for ADG on the first and second day after weaning (Fig. 5b). Maternal feed restriction improved ADG between d0–2 post-weaning (RES: 128 ± 17 vs. FF: −32 ± 21 g/day; *P* < 0.0001) and tended to improve ADG over the two weeks after weaning (RES: 283 ± 9 vs. FF: 263 ± 9 g/day; Table 3). RES-piglets did not differ anymore in BW from FF-piglets at d15 post-weaning (RES: 10.18 ± 0.17 vs. FF: 10.43 ± 0.14 kg; Table 3). The play-feeder improved ADG between d0–2 (PL: 91 ± 24 vs. CON: 6 ± 27 g/day; *P* = 0.001), d2–5 (PL: 262 ± 12 vs. CON: 222 ± 12 g/day), d5–15 (PL: 342 ± 10 vs. CON: 313 ± 7 g/day; Fig. 5b) and during the two weeks after weaning (PL: 293 ± 8 vs. CON: 253 ± 7 g/day; Table 3). Consequently, PL-piglets had a higher weight than CON-piglets at d15 post-weaning (PL: 10.57 ± 0.13 vs. CON: 10.04 ± 0.16 kg; Table 3). The treatments did not affect FCR (Table 3). The play-feeder resulted in a shorter duration of diarrhoea (PL: 1.72 ± 0.37 vs. CON: 3.00 ± 0.47 days of diarrhoea during 2 weeks post-weaning), a lower FCS (PL: 0.13 ± 0.03 vs. CON: 0.26 ± 0.04) and a lower prevalence of watery diarrhoea in the weaner pens in the first two weeks post-weaning (PL: 16.7 vs. CON: 61.1% of pens; Table 3). SF did not affect the faecal consistency parameters after weaning.

## Discussion

A low lactational feeding level of the sow was used to reduce the energy intake of piglets from milk and to investigate whether creep feed intake is homeostatically driven, while a play-feeder was used to present creep feed in an explorative and playful context and to study whether creep feed consumption is exploratory driven. Maternal feed restriction motivated piglets to eat creep feed and thereby eased the weaning transition, as reflected by a higher feed intake and growth and less body lesions, mainly in the first two days after weaning. Although the play-feeder did not improve creep feed intake of piglets, it stimulated feeder exploration before weaning and had a broad and long-term beneficial effect on ingestive behaviours, feed intake, growth, faecal consistency and body lesions and damage in the two weeks after weaning.

Consistent with predictions, maternal feed restriction by 50% resulted in a 16% lower ADG of piglets during lactation and a 12% lower weaning weight. Previous studies showed that a low lactational feeding level of the sow reduced energy supply from milk, due to a lower milk production and changes in milk composition^42,43^, resulting in a lower gain of piglets^11,42,43^. Indeed, RES-sows used in this study had a lower calculated milk production and a lower milk fat percentage, as determined by milk sample analysis (Costermans *et al*., submitted). Litters of RES-sows consumed three times as much feed during the last two days before weaning, doubled their time spent eating one day before weaning and had double as much eaters at weaning compared to litters of FF-sows. These findings disagree with the results of Sulabo *et al*.^11^, in which maternal feed restriction stimulated creep feed intake only during the first days after its implementation and also did not stimulate more piglets to eat creep feed; however, in their study RES-sows were fed 25% less than FF-sows and therefore weaning weight was only 6% less. Hunger as homeostatic drive was likely the biggest motivator for RES-piglets to eat creep feed. Alternatively, it cannot be excluded that potential behavioural changes of the sow occurring as result of feed restriction^44–46^ may have influenced the eating behaviour of RES-piglets. Our results support that the compensatory feeding hypothesis at least holds on litter level, as RES-piglets grew less during lactation and consumed more creep feed towards weaning. Even though creep feed consumption by RES-piglets was only enhanced for a short period (from two days prior to weaning) and by a small amount (34 g/piglet) and creep feed intake was still generally low by then (25 g/piglet/d), RES-piglets did eat 60 g/d more in the first five days post-weaning and did grow 160 g/d faster in the first two days post-weaning (when they ate 83 g/d more) compared to piglets reared by FF-sows. RES-litters had 22% more eaters of creep feed at weaning than FF-litters, which may also have stimulated post-weaning feed intake via social learning, as non-eaters can learn about solid feed from eaters^47^. However, as the ADG of RES-piglets in the first two days after weaning was two times larger than the ADFI of RES-piglets in the same period, there seems to be an effect on growth beyond the energy intake from feed. Piglets may have experienced lower levels of stress and/or neophobia due to a more gradual dietary change^48^. Moreover, RES-piglets had fewer body lesions than FF-piglets within CON after mixing post-weaning and spent less time ear biting at 2 weeks post-weaning than FF-piglets. A lower time spent biting pen mates and a lower number of injuries from aggression might also be related to a reduced level of stress^49–51^, but RES-piglets may also have fought less after mixing to save energy and, instead, engage in exploratory and ingestive behaviour shortly after weaning. Benefits of creep feed intake during lactation on feed intake and growth of piglets in the initial weaner period have been demonstrated previously^8–11^. Getting piglets to eat creep feed is therefore important to reduce the post-weaning dip in performance. We showed that the energy intake of piglets from milk is one of the fundamental factors that influences their creep feed intake, but we do not recommend maternal feed restriction as a feeding strategy to improve creep feed intake and to facilitate the weaning transition of piglets, as feed restriction is detrimental for piglet welfare^52^, sow welfare^44^ and sow future reproductive performance^11,37,53^. This study illustrated that RES-piglets were able to compensate growth once the nutritional restrictions were eliminated at weaning, as RES-piglets attained the BW of FF-piglets at two weeks post-weaning.

In line with our prediction, the play-feeder was successful in eliciting and sustaining exploratory behaviour in suckling piglets, as the time spent on exploration towards the feeder was increased by 3.5 to 6 times, as well as the number of piglets visiting the feeder by 24 to 34%. Thereby, the play-feeder in our study seemed to stimulate exploratory behaviour more elaborately than the exploration-stimulating feeder of Kuller *et al*.^29^, probably because it allowed object play, object manipulation (e.g. objects were chewable, moveable) and a larger repertoire of foraging behaviours. We found that time spent exploring the feeder correlated with time spent eating (at d9, 16 and 23) and with feed intake (at d9 and 16, but not at d23), suggesting that exploratory behaviour may be mainly important in familiarizing piglets with creep feed before actual ingestion starts. Yet, we could not confirm a clear positive effect of the play-feeder on creep feed intake on litter level, as was observed by Kuller *et al*.^29^, as time eating creep feed, the amount of feed consumed and the percentage of eaters did not significantly differ between the creep feed presentation strategies. However, the play-feeder increased the number of piglets in better eater classes, but only when reared by RES-sows. Moreover, RES-PL ate the most during the first four hours after weaning and grew the fastest during the first day after weaning compared to the other three treatment groups. In addition, FF-CON had more body lesions due to aggressive interactions after mixing than RES-CON and tended to have more lesions than RES-PL and FF-PL. This corresponds to the observation that FF-CON also had significantly the lowest ADG on the second day after weaning compared to the other three groups. The play-feeder thus seemed to attract especially the piglets of sows that had a low lactational feed intake and may therefore be particularly useful in practise to support litters of sows with poor milk production due to e.g. illness or heat stress.

Despite the play-feeder only subtly affected individual creep feed classification compared to feed restriction of the sow, the play-feeder showed greater, broader and longer-term beneficial post-weaning effects, as ingestive behaviours, feed intake and growth of PL-piglets were significantly improved by 15% for (at least) two weeks post-weaning, resulting in a 5% higher weight at d15 post-weaning. The play-feeder also reduced the prevalence (by 44%), duration (by 1.3 days) and severity of diarrhoea (by halving the FCS), and decreased the number of body lesions (by 35%), piglets with ear damage within FF, and piglets with tail damage within RES at d15 post-weaning. A similar effect was observed by Telkänranta *et al*.^54^ in which growing pigs with pre-weaning experience of ropes and paper had a reduced prevalence of severe tail damage. It has been hypothesized that a higher feed intake and growth, an increase in faecal consistency and a decrease in maladaptive behaviour of weaner piglets are related to each other and may all result from lower levels of stress and/or neophobia at weaning^48,55,56^. Firstly, PL-piglets might have been better acquainted with the sight and smell of the nursery feed (that was mixed with the creep feed during the last two days prior to weaning) compared to CON-piglets, as result of a higher feeder visiting time and more piglets visiting, subsequently resulting in reduced neophobia and a higher intake of that feed after weaning^57^. Secondly, we speculate that PL-piglets might have developed a positive association between (the smell of) solid feed and object play. Social play has a pleasurable and rewarding nature through opioid, endocannabinoid, dopamine and noradrenaline systems^58^ and it has been suggested that object play may also be self-rewarding^59^. Thirdly, we predict that the higher frequency of manipulating behaviours on the toys of the play-feeder, such as chewing, might have facilitated the transition from exploratory to eating behaviour (e.g. via stimulation of mastication muscles^60,61^, changes in gut hormone secretion^62^ and reward circuits responses^63^ or habituation to a dry mouthfeel), but only became evident when their need to consume solid feed increased, either by weaning or maternal feed restriction. Lastly, the provision of play-objects before weaning, thereby eliciting an early play experience for piglets, might also improve their ability to emotionally cope with weaning according to the ‘training for the unexpected hypothesis’^64^. However, the latter hypothesis alone seems insufficient to explain the broad effects we found on piglet’s ability to deal with the weaning process, as providing (non-edible) play-objects in the farrowing pen as early life enrichment (of which cotton ropes are mostly studied) only limitedly affected later life performance^31,54,65^. That is why we expect the association between exploration/play and feed to be important in eliciting beneficial effects and therefore recommend play-objects to be located at the feeder instead of else in the farrowing pen. Using play-objects at the creep feeder showed positive effects for the welfare of piglets, as it increased the frequency of normal exploratory and object play behaviour and increased their ability to cope with weaning in a broad sense, despite that the play-feeder was only provided before weaning. We predict the beneficial effect of the play-feeder on post-weaning adaptation to be even more substantial when the play-feeder is also provided after weaning, however this remains to be studied.

In conclusion, litters reared by restrictedly-fed sows grew less, had more eaters and ate more before weaning, suggesting that pre-weaning feed intake is at least partly homeostatically driven. This pre-weaning acquaintance with feed improved feed intake and growth in the first days after weaning. The play-feeder, which was only provided before weaning, encouraged piglets to explore and play with (the toys of) the feeder and sustained piglets’ interest up to weaning. The main finding of this study was that the play-feeder had no effect on creep feed intake in itself, but remarkably eased the weaning transition, as reflected in improved post-weaning feed intake and growth and reduced diarrhoea and body damage. This implies that providing toys at the creep feeder can be considered as an easy applicable enrichment and feeding strategy to reduce weaning stress and improve the health, welfare and productivity of piglets around weaning. The mechanism(s) underlying this beneficial effect are not yet well understood and deserve further attention.

## Supplementary information

Supplementary Table S2, S3 and S4

Supplementary Video S1

## Data Availability

The datasets generated and analysed during the current study are available from the corresponding author on request.
