# Supplementary material for: Feed intake of the sow and playful creep feeding of piglets influence piglet behaviour and performance before and after weaning
Source: Sci Rep. 2019 Nov 6;9:16140. doi: 10.1038/s41598-019-52530-w (PMC6834851; doi:10.1038/s41598-019-52530-w)
Supplement: Supplementary file 1 — Supplementary Table S2, S3 and S4 [file 41598_2019_52530_MOESM1_ESM.docx]

**Feed intake of the sow and playful creep feeding of piglets influence piglet behaviour and performance before and after weaning**

Anouschka Middelkoop^1^, Natasja Costermans^1,2^, Bas Kemp^1^, J. Elizabeth Bolhuis^1,*^

^1^ *Adaptation Physiology Group, Department of Animal Sciences, Wageningen University & Research, P.O. Box 338, 6700 AH Wageningen, The Netherlands*

^2^ *Human and Animal Physiology Group, Department of Animal Sciences, Wageningen University & Research, P.O. Box 338, 6700 AH Wageningen, The Netherlands*

*[liesbeth.bolhuis@wur.nl](mailto:liesbeth.bolhuis@wur.nl)

**Supplementary Table S2.** Nutrient profile of the creep feed

| **Calculated nutrient composition^1^** | **Creep feed** |
| --- | --- |
| Dry matter | 891 |
| Starch | 290 |
| Non-starch polysaccharides^2^ | 261 |
| Crude protein | 195 |
| Crude fat | 61 |
| Crude fibre | 44 |
| Crude ash | 57 |
| Calcium | 9.1 |
| Phosphorus | 6.1 |
| Sodium | 2.2 |
| Standardized ileal digestible lysine | 11.9 |
| Standardized ileal digestible methionine | 4.8 |
| Standardized ileal digestible threonine | 7.1 |
| Standardized ileal digestible tryptophan | 2.4 |
| Net energy | 11.8 |

^1^ According to CVB (2007). Nutrients are presented in g/kg dry matter, except for dry matter (g/kg) and net energy (MJ/kg).

^2^ Calculated as the difference between dry matter and the sum of starch, sugars, crude protein, crude fat and crude ash.

**Supplementary Table S3.** Ingredient composition of the creep feed

| **Ingredient component** | **%** |
| --- | --- |
| Wheat | 21.9 |
| Barley | 15 |
| Maize | 15 |
| Soy protein concentrate | 7 |
| Soybeans (heat treated) | 5 |
| Galacto-oligosaccharides | 5 |
| Potato protein | 4 |
| Sugarbeet pulp (dehydrated) | 4 |
| Oat hulls | 4 |
| Inulin | 4 |
| High-amylose starch (± 75% amylose) | 4 |
| Soybean oil | 3 |
| Blood meal (spray dried) | 2 |
| Dicalcium phosphate | 1.7 |
| Sucrose | 1.5 |
| Calcium carbonate | 1.0 |
| Sodium chloride | 0.5 |
| Premix^1^ | 0.5 |
| Potassium bicarbonate | 0.3 |
| L-lysine hydrochloride | 0.3 |
| DL-methionine | 0.2 |
| L-threonine | 0.04 |
| L-tryptophan | 0.04 |
| **Total** | **100** |

^1^ Vitamin and mineral premix (per kg of feed): vitamin A: 10000 IU, vitamin D3: 2000 IU, vitamin E: 40 mg, vitamin K: 1.5 mg, vitamin B1: 1 mg, vitamin B2: 4 mg, vitamin B6: 1.5 mg, vitamin B12: 0.02 mg, niacin: 30 mg, D-pantothenic acid: 15 mg, choline chloride: 150 mg, folate: 0.4 mg, biotin: 0.05 mg, iron: 100 mg, copper: 20 mg, manganese: 30 mg, zinc: 70 mg, iodine: 0.7 mg, selenium: 0.25 mg, anti-oxidant: 125 mg.

**Supplementary Table S4.** Behaviours of piglets after weaning

| Behaviour | | Description |
| --- | --- | --- |
| ‘Ingestive behaviour‘ | | |
|  | Eating feed | Eating or chewing feed (at the feeder) |
|  | Drinking | Drinking water from drinking trough |
| ‘Exploratory behaviour’ | | |
|  | Exploring feeder | Sniffing, touching (with snout), rooting or chewing on feeder |
|  | Exploring feed | Sniffing, touching (with snout) or rooting the feed in the feeder |
|  | Exploring drinking trough | Sniffing, touching (with snout) or chewing on drinking trough |
|  | Nosing environment | Sniffing, touching (with snout) part of the pen (e.g. floor, wall) |
|  | Rooting environment | Rooting part of the pen, scraping floor with one the front legs |
|  | Chewing environment | Chewing or nibbling part of the pen, including toy |
|  | Chewing air | Chewing air (not chewing on part of the pen, feed or toy) |
| ‘Postures and locomotion’ | | |
|  | Lying eyes closed | Lying on side or belly with eyes closed without performing any other described behaviour |
|  | Lying eyes open | Lying on side or belly with eyes open, sitting or kneeling without performing any other described behaviour |
|  | Standing | Piglet is upright, standing, without performing any other described behaviour |
|  | Walking | Piglet is walking, without performing any other described behaviour |
| ‘Play behaviour’ | | |
|  | Playing individually | Play activities that involve one player; running across pen, rolling, pivoting, tossing head, flopping, sliding, scampering, nudging |
|  | Playing socially | Play activities that involve more players; e.g. running, pivoting, scampering, sliding across pen together, play fighting. |
|  | Playing with chew object | (Energetically) shaking head with chew object in mouth |
| ‘Pig-directed behaviour’ | | |
|  | Nosing body | Sniffing, touching (with snout) part of the body of a pen mate excluding snout |
|  | Snout contact | Mutual snout contact with pen mate |
|  | Ear biting | Chewing, nibbling or sucking the ear of a pen mate |
|  | Tail biting | Chewing, nibbling or sucking the tail of a pen mate |
|  | Belly nosing | Rubbing belly of a pen mate with up and down movements of the snout or sucking the navel or skin of the abdominal area of a pen mate |
|  | Manipulating pen mates | Chewing, nibbling or sucking part of the body of a pen mate excluding ear, tail and abdominal area |
|  | Mounting pen mates | Standing on hind legs while having front legs on pen mate |
|  | Aggression | Aggressively ramming, pushing, head-knocking, lifting or biting a pen mate, including mutual fighting |
| ‘Other behaviour’ | | |
|  | Comfort | Rubbing body against objects or pen mates, scratching body with hind legs or stretching (part of) body |
|  | Eliminating | Defecating or urinating |
